# Divalent Aptamer-Functionalized Nanochannels for Facile Detection of Cancer Cell-Derived Exosomes

**DOI:** 10.3390/s23229139

**Published:** 2023-11-13

**Authors:** Yue Huang, Fangfang Zhou, Fengjie Jia, Nana Yang

**Affiliations:** 1 Department of Food Science and Engineering, College of Light Industry and Food Engineering, Nanjing Forestry University, Nanjing 210037, China; 2 State Key Laboratory of Analytical Chemistry for Life Science, School of Life Sciences, Nanjing University, Nanjing 210023, China; 3 Department of Obstetrics and Gynecology, The First Affiliated Hospital of Nanjing Medical University, Nanjing 210029, China

**Keywords:** electrochemical biosensor, divalent, nanochannels, facile, cancer cell-derived exosomes

## Abstract

Exosomes are considered potential biomarkers for early screening and accurate non-invasive diagnosis of cancer, so development of innovatively facile approaches for the detection of cancer cell-derived exosomes has become increasingly important. Herein, we propose a facile electrochemical biosensor based on divalent aptamer-functionalized nanochannels for highly efficient detection of cancer cell-derived exosomes. The aptamer against transmembrane receptor protein CD63 and the aptamer targeting membrane protein EpCAM are simultaneously immobilized on the nanochannels to construct the divalent aptamer-functionalized nanochannels. Thus, the target exosomes can be recognized and selectively captured by the functionalized nanochannels in a divalent collaborative manner. The combined exosomes overlay the ion channel effectively and hinder the ionic flow through the nanochannels, resulting in an evidently varied ionic transport behavior corresponding to the abundance of exosomes. The divalent aptamer-functionalized nanochannels can substantially promote the binding stability and enhance the detection specificity, while the sensitivity of detection is improved greatly by virtue of the amplified response of array channels synergized with the electrochemical technique. Therefore, the developed biosensor provides a highly specific, sensitive, and accurate approach for the detection of cancer cell-derived exosomes, which may hold great potential for application in early clinical cancer diagnosis.

## 1. Introduction

Exosomes are nanoscale extracellular vesicles excreted by many cells, carrying plenty of contents involving nucleic acid, membrane proteins, small metabolites, and so on [1,2,3]. As crucial communication tools, exosomes participate in the inter-cell substance and information communication and play a vital role in the regulation of physiological and pathological processes [4,5]. An increasing amount of evidence indicates that cancer cell-derived exosomes are closely correlated with tumor initiation, progression, and migration [6]. Cancer cell-derived exosomes exist widely in biofluids like blood, saliva, and urine and may show unnormal expression levels in the early stage of cancer [7]. In addition, owing to the protective effect of the phospholipid bilayer, exosomes can be intrinsically stable and easy to store long-term for further separation and detection [8]. Therefore, exosomes have been considered reliable and valuable biomarkers for early screening and accurate non-invasive diagnosis of cancer. Accordingly, development of innovatively facile methods for the detection of cancer cell-derived exosomes has become increasingly important. However, due to the diverse origins of parent cells, heterogeneous physical size, and low abundance of exosomes in biofluids [9], efficient and accurate analysis of cancer cell-derived exosomes still remains a challenge. The presently available methods to detect exosomes such as flow cytometry, enzyme-linked immunosorbent assay, microfluidics, fluorescence spectroscopy, etc. usually require complicated designs and laborious operation or sophisticated equipment or cannot meet the satisfactory sensitivity, which may limit the wide applications of these methods [10,11,12,13]. Therefore, further efforts to develop novel methods with reliable detection performance for cancer cell-derived exosomes are yet greatly in demand.

Porous anodic alumina (PAA) films have gained much interest for the construction of functionalized nanochannels and the development of ingenious biosensors [14]. The tiny conduction channels in the barrier layer of PAA films possess a controllable ion transport property and high switching efficiency [15,16]. As electrochemical biosensing platforms, the nanochannels in PAA films can not only decrease the background noise but also allow signal amplification of several orders of magnitude, contributing to high detection sensitivity and accuracy [17,18]. Therefore, a series of biosensors based on the nanochannel arrays of PAA films have been constructed for the flexible detection of multiple targets, ranging from small molecules to micrometer-scale cells. For example, Li’s group successfully achieved the sensitive detection of Hg^2+^ and SARS-CoV-2 by using DNA-functionalized PAA films [19,20]. Cao et al. reported an electrochemical biosensor to efficiently capture and sensitively detect circulating tumor cells based on aptamer-modified PAA [21]. However, up to now, studies making use of nanochannels for the detection of exosomes have been rarely reported.

**Scheme 1:**
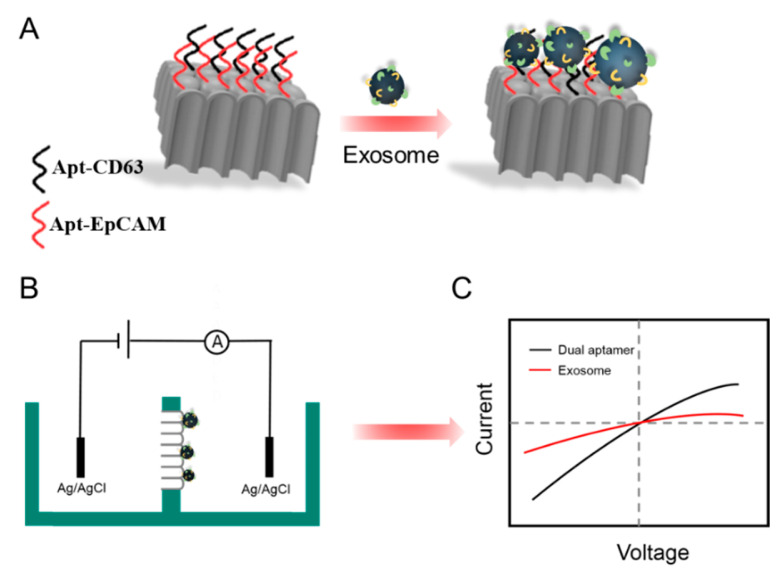
(**A**) Principle of the divalent aptamer-functionalized nanochannels for detection of exosomes. (**B**) Schematic representation of the electrochemical detection device. (**C**) Diagrammatical illustration of the altered electrochemical responses towards exosomes’ presence and absence.

Aptamers are single-strand nucleic acid oligomers termed “chemical antibodies” with high affinity and specificity towards various targets; they also feature the superiorities of low molecular weight, easy synthesis and modification, low immunogenicity, good stability, and reusability [22,23,24]. On the other hand, exosomal proteins, such as CD63, CD9, TSG101, and EpCAM, reflecting significant information about the cancer microenvironment, are highly enriched in exosomes derived from cancer cells [25,26]. Hence, these membrane proteins can serve as aptamers against markers in the analysis of cancer cell-derived exosomes. Herein, we propose a facile electrochemical biosensor for highly specific and sensitive detection of cancer cell-derived exosomes based on divalent aptamer-functionalized nanochannels. As shown in Scheme 1A, the aptamer specific to transmembrane receptor protein CD63 (Apt-CD63) and the aptamer aiming at membrane protein EpCAM (Apt-EpCAM) are simultaneously immobilized on the barrier layer of PAA film to construct the divalent aptamer-functionalized nanochannels. Thus, target exosomes can be captured selectivity through dual identification. The captured exosomes will cover the array channels’ surface efficiently, hindering the ion flow through the nanochannels. The prepared PAA film is fixed in the middle of the self-made H-type electrolytic cell (Scheme 1B), and the ion transport behavior is monitored by the current-voltage (*I*–*V*) characteristics (Scheme 1C). As a result, an evidently varied ion transport response can be observed corresponding to the abundance of exosomes. The divalent aptamer-functionalized nanochannels provide dual collaborative recognition sites that can substantially improve the capture efficiency and enhance the binding stability, facilitating the highly specific and accurate detection of cancer cell-derived exosomes. In addition, by relying on the amplification effect of array channels synergized with the electrochemical technique, sensitive detection of exosomes can be accomplished in one step without lengthy experimental processes or intricate designs of nucleic acid sequences. Therefore, the electrochemical biosensor based on divalent aptamer-functionalized nanochannels enables highly specific, sensitive, and accurate detection of cancer cell-derived exosomes. Moreover, the biosensor exhibits commendable analytical capability in real serum samples, holding promise in applications for early clinical diagnosis of cancer.

## 2. Materials and Methods

### 2.1. Materials and Reagents

HeLa cells were obtained from Shanghai Institutes for Biological Sciences (Shanghai, China). The (3-Aminopropyl)triethoxysilane (APTES), penicillin-streptomycin solution, Dulbecco’s modified Eagle’s culture medium (DMEM), and ethanol were bought from Sigma-Aldrich (Shanghai, China). The PAA films were ordered from Hefei Puyuan Nanotechnology Co., Ltd. (Anhui, China). Potassium chloride (KCl) was ordered from Nanjing Chemical Reagent Co., Ltd (Nanjing, China). All oligonucleotides listed in Table S1 were custom synthesized by Sangon Biotech Co., Ltd. (Shanghai, China). All solutions were prepared using ultrapure water (18.2 MΩ cm) purified with a Milli-Q system (Bedford, MA, USA).

### 2.2. Cell Culture and Exosomes Purification

DMEM with high glucose containing 10% (*v*/*v*) fetal bovine serum (FBS) and 1% (*v*/*v*) penicillin-streptomycin was employed to culture HeLa cells in a humidified atmosphere (with 5% CO_2_) at 37 °C. As the cells proliferated to the 70% confluency, we deserted the medium and slightly washed the cells twice with phosphate-buffered saline (PBS). After culturing the cells for 48 h in DMEM with exosome-free FBS, the cell medium supernatant was finally gathered to extract exosomes.

The collected medium supernatant was centrifuged for 20 min at 3000× *g* to exclude large particles such as cell debris and then centrifuged at 10,000× *g* for 30 min to eliminate the microvesicles. The cell supernatant was then filtered by using a 0.22 μm syringe filter, and the resulting solution was subsequently centrifuged for 2 h at 100,000× *g*. The obtained exosomes sediment was finally resuspended using PBS and deposited at −80 °C.

### 2.3. Functionalization of PAA Film and Detection of Exosomes

The pretreatment procedures of PAA film and preparation of APTES-modified PAA film were conducted according to previous report [20]. Then a 20 μL mixture solution of CHO-labeled Apt-EpCAM and CHO-labeled Apt-CD63 (*v*/*v* = 1:1, 40 μM in PBS) was dispersedly dropped onto the APTES-modified PAA film and reacted at 25 °C for 24 h. The PAA film was hung in a sealed glass bottle with a little water at the bottom to prevent evaporation of the aptamer solution. Then the PAA film was rinsed with distilled water to eliminate the residual aptamers, following which the PAA film was incubated with 10 mg/mL bovine serum albumin (BSA) solution for 1 h to prevent nonspecific adsorption. Finally, the PAA film was washed with ultrapure water and deposited at 4 °C for usage.

To detect HeLa cell-derived exosomes, an exosome solution (20 μL) of varying concentrations was added onto the divalent aptamer-functionalized PAA film and reacted for 2 h at 37 °C to allow the capture of exosomes. Afterward, the PAA film was washed with PBS buffer and ready for electrochemical measurements.

### 2.4. Characterization of Exosomes and PAA Films

Nanoparticle tracking analysis (NTA) and transmission electron microscopy (TEM, Hitachi H-7650, Tokyo, Japan) were employed to characterize the prepared exosomes. Scanning electron microscope (SEM, SU8020, Hitachi, Tokyo, Japan) was used to observe the morphology of bare PAA film. X-ray photoelectron spectroscopy (XPS, ESCALAB 250XI, Thermo Fisher Scientific, Waltham, MA, USA) was employed to characterize the functionalization procedure of PAA film. The exosome-combined PAA film was characterized by atomic force microscopy (AFM, Bruker, Mannheim, Germany).

### 2.5. Electrochemical Measurement

Electrochemical linear sweep voltammetry (LSV) was employed for *I*–*V* measurements, performed in 10 mM KCl solution (pH 7.4) by a CHI 660D electrochemical workstation. The prepared PAA film was placed in the middle of the homemade H-type electrolytic tank with two Ag/AgCl electrodes (0.6 mm, in diameter) as the cathode and anode, respectively. Cations (K^+^ ions) and anions (Cl^−^ ions) in the electrolyte solution were actuated to move directionally when voltage biases were applied to the asymmetric nanochannels through two nonpolarized Ag/AgCl electrodes, leading to the ionic current rectification [27]. *I*–*V* properties between −1.2 and +1.2 V were recorded at a scan rate of 0.05 V/s.

## 3. Results and Discussion

### 3.1. Characterization of Exosomes

**Figure 1:**
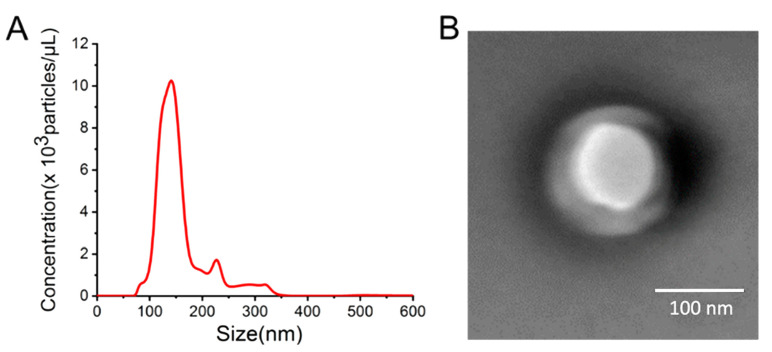
Characterization of HeLa cell-derived exosomes: the NTA result (**A**), the TEM image (**B**).

To prove the successful extraction of exosomes from HeLa cells, NTA and TEM were employed to measure the concentration and the typical morphology of exosomes. The NTA result (Figure 1A) shows that the HeLa cell-derived exosomes possess an average hydrodynamic diameter of approximately 140 nm with a concentration of 1.33 × 10^10^ particles/mL. The typical morphology of exosomes observed from TEM (Figure 1B) displays as roundness or an ellipse with a diameter consistent with the result of NTA.

### 3.2. Characterization and Functionalization of the PAA Film

**Figure 2:**
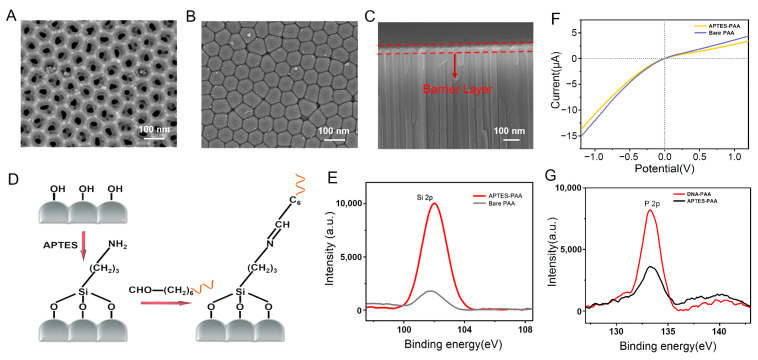
Functionalization and characterization of the PAA film. SEM images of the PAA film: porous layer (**A**), barrier layer (**B**), and flank view (**C**). (**D**) Diagram of the functionalization procedure for PAA film. (**E**) XPS measurement for Si 2p to characterize the bare PAA film (gray curve) and the immobilization of APTES (red curve). (**F**) *I*–*V* measurement to characterize the bare PAA film (purple curve) and the immobilization of APTES (yellow curve). (**G**) Characterization of the immobilizations of APTES (black curve) and dual aptamers (red curve) on PAA film by XPS measurement for P 2p.

The characteristic morphology of the prepared PAA film is determined by SEM. Figure 2A exhibits the porous layer of PAA film with array channels with diameters of approximately 50 nm. Small hexagonal structure with regular arrangement is shown in the barrier layer of PAA film (Figure 2B). Owing to the very small size down to sub-nanometers, ion channels in the barrier layer cannot be viewed from the SEM image. Figure 2C shows the side view of the PAA film with the marked barrier layer. The functionalization procedure of PAA film is illustrated in Figure 2D, while XPS is used to examine the procedure. As shown in Figure 2E, compared to the XPS spectrum of bare PAA film (gray curve), a distinct Si 2p peak appears after treatment with APTES (red curve), which is ascribed to the formation of –Si–O–, indicating the successful immobilization of APTES on the PAA film. In addition, the introduction of APTES is verified through the evaluation of the *I*–*V* characteristic. Owing to the asymmetric nanochannel of PAA film and its surface modification, obvious ionic current rectification can be obtained. The current at one polarity voltage is distinctly lower or bigger than that at the opposing polarity voltage in ionic current rectification, resulting in the asymmetric *I*–*V* curves [20,27]. With 10 mM KCl as an electrolyte solution, two Ag/AgCl electrodes are employed to apply the potential and measure the ion current. Figure 2F shows that the ion current decreases after modification with APTES. This is because the introduction of APTES reduces the wettability of the PAA film, which can influence the ion flow through the nanochannels. The functionalization of dual aptamers on the PAA film is verified by specified XPS tests for P 2p. As displayed in Figure 2G, an obviously increased P 2p peak emerges after the attachment of dual aptamers carrying plenty of phosphodiesters, suggesting the successful construction of the divalent aptamer-functionalized nanochannels.

### 3.3. Feasibility Investigation

**Figure 3:**
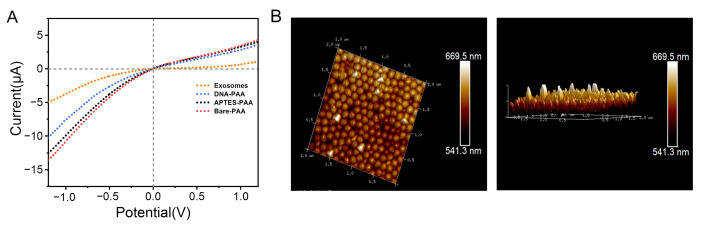
Feasibility verification of the biosensor. (**A**) *I*–*V* properties of the bare PAA film (red dots), PAA film modified with APTES (black dots), divalent aptamer-functionalized PAA film before (blue dots) and after (yellow dots) incubation with the exosomes. (**B**) AFM image of the divalent aptamer-functionalized PAA film after incubation with the exosomes.

We have first examined the feasibility of the biosensor through the *I*–*V* measurement by using the LSV. Little changes on the surface of PAA film can bring about a significant current variation. As shown in Figure 3A, a clear decrease of the current value at −1.2 V appears after modification with dual aptamers, because the steric hindrance increasement induced by the dual aptamers would impede the ion transport. The specific combination with the target exosomes further increases the steric hindrance, resulting in an evident current drop. In addition, the feasibility for exosome detection has also been intuitively verified by the AFM. Distinct profiles of the nanochannels and exosomes (white bulges) are shown in Figure 3B, and the increased thickness reveals the successful capture of target exosomes. According to the alteration of the ion transport property reflected by the *I*–*V* measurement results and the AFM imaging data, the newly developed biosensor based on the divalent aptamer-functionalized nanochannels is feasible for the detection of exosomes.

### 3.4. Optimization of Experimental Conditions

The key experimental conditions of the biosensor are studied to obtain the optimal performance for exosome detection. The concentration of dual aptamers to functionalize the nanochannels is closely related with the capture efficiency of exosomes, so the optimal concentration of dual aptamers is investigated. The ratio (|(I − I_0_)/I_0_|, I, and I_0_ represent the current values at −1.2 V with the existence and absence of target exosomes respectively) versus the concentration of aptamer is shown in Figure S1. It is found that the ratio changes with the aptamer concentration increasing from 0 to 50 μM and reaches the biggest value at 40 μM. Thus, 40 μM is considered to be sufficient to accommodate the exosomes. On the other hand, the proper mixture ratio of Apt-CD63 and Apt-EpCAM is studied. As shown in Figure S2, the value of |(I − I_0_)/I_0_| alters with the variation of the volume ratio of Apt-CD63 to Apt-EpCAM and gets the maximum at the volume ratio of 1:1. Thus, the optimal volume ratio of Apt-CD63 to Apt-EpCAM is set as 1:1. Besides, in order to improve the detection efficiency and accuracy, the incubation time with exosomes is optimized. Figure S3 shows that the ratio (|(I − I_0_)/I_0_|) first increases sharply when the incubation time increases from 30 to 120 min and then decreases slightly with the extension of time. The results indicate that the exosomes combination can be completed after 120 min, while too long of an incubation time may disrupt the structure of exosomes. Therefore, 120 min is adopted as the optimum incubation time of exosomes for the experiments.

### 3.5. Investigation of Detection Performance

**Figure 4:**
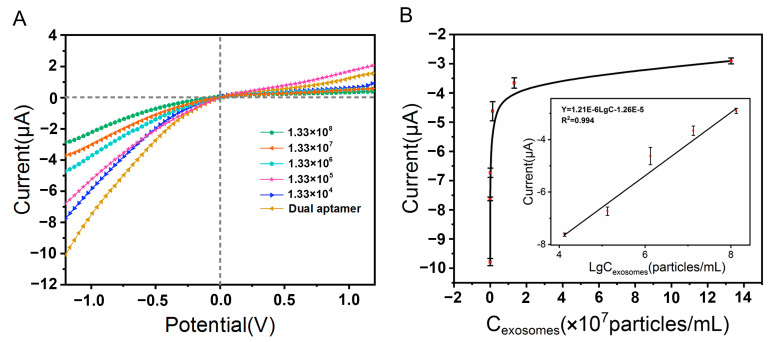
Quantitative detection of the HeLa cell-derived exosomes. (**A**) *I*–*V* properties for detecting various concentrations of HeLa cell-derived exosomes. (**B**) The relationship between the current value at −1.2 V and the exosome concentration. Insert: linear calibration plot of the current value versus the logarithm of exosome concentration. Error bars are the standard deviations of three parallel measurements.

The biosensor is employed to determine the quantities of HeLa cell-derived exosomes with the optimum experimental conditions. The *I*–*V* properties reflecting the changed ionic transfer behaviors of the divalent aptamer-functionalized nanochannels after incubation with different concentrations of exosomes are shown in Figure 4A, and the correlation between the current value at −1.2 V and the exosome concentration is depicted in Figure 4B. The results reveal that the absolute current value measured at −1.2 V decreases continuously as the increasement of exosome concentration. The Figure 4B insert shows a linear response range of 1.33 × 10^4^~1.33 × 10^8^ particles/mL of the biosensor for the HeLa cell-derived exosomes detection. The limit of detection is estimated to be 4.43 × 10^3^ particles/mL, suggesting admirable sensitivity of this biosensor.

**Figure 5:**
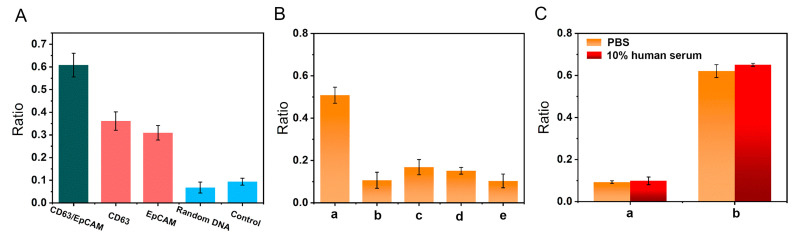
(**A**) Current changes of nanochannels functionalized with dual aptamers, Apt-CD63, Apt-EpCAM, and random DNA after incubation with exosomes of 1.33 × 10^7^ particles/mL. Control: bare PAA film. (**B**) Selectivity investigation of the biosensor. (a) exosomes, (b) HeLa cell debris, (c) 231 cell debris, (d) liposomes, (e) blank. The concentrations of HeLa cell-derived exosomes and the other three analytes are 1.33 × 10^6^ particles/mL and 1.33 × 10^8^ particles/mL, respectively. Blank: 1 × PBS. (**C**) Analytical performance of the developed biosensor in PBS and 10% human serum: (a) without exosomes, (b) with 1.33 × 10^7^ particles/mL exosomes. Error bars are the standard deviations of three parallel measurements. Ratio represents |(I − I_0_)/I_0_|; I is the current value at −1.2 V with the existence of HeLa cell-derived exosomes, and I_0_ is the current value at −1.2 V with the absence of HeLa cell-derived exosomes.

To investigate the specificity and recognition efficiency of divalent aptamer-functionalized nanochannels for exosomes, sole Apt-CD63, Apt-EpCAM, and random DNA are employed to functionalize the nanochannels for the detection of exosomes. As shown in Figure 5A, with the same concentration of exosomes, the current response produced by using divalent aptamer-functionalized nanochannels is much higher than that produced by using nanochannels functionalized with sole aptamer. Meanwhile, the current response of random DNA-functionalized nanochannels is similar to that of the bare PAA film. The results indicate that the collaboration of dual aptamers enables the efficient recognition and capture of target exosomes, endowing the biosensor with excellent specificity and accuracy. Besides, to further test the selectivity of the divalent aptamer-functionalized nanochannels for HeLa cell-derived exosomes, other kinds of analytes are detected by the proposed biosensor, and the results are presented in Figure 5B. It is found that the ion current varies significantly in the case of the detection of HeLa cell-derived exosomes; as for cell debris and liposomes, no special recognition occurs between dual aptamers and these analytes, and accordingly, no obvious changes of ionic current appear, which stays very similar with the blank. Thus, it can be inferred from the results that the proposed biosensor can detect HeLa cell-derived exosomes with prominent selectivity.

To validate the detection performance of the developed biosensor in an intricate matrix, HeLa cell-derived exosomes are spiked in 10% human serum and detected by the biosensor. As shown in Figure 5C, compared to the current signals obtained from the same concentration of exosomes in PBS, the current responses in 10% human serum reveal negligible distinction. The results suggest that potential interfering substances in human serum actually produce little influence on the biosensor due to the high specificity and the simple one-step operation. Therefore, the developed biosensor can perform well with complicated biological components, indicating a potential application in clinical samples.

## 4. Conclusions

In summary, we have developed a novel electrochemical biosensor based on divalent aptamer-functionalized nanochannels for highly specific, sensitive, and accurate detection of cancer cell-derived exosomes. The divalent aptamer-functionalized nanochannels can recognize and capture the target exosomes selectively in a divalent collaborative manner, endowing the biosensor with excellent specificity and accuracy. On the other hand, in virtue of the unique amplified response of array channels orchestrated with the electrochemical technique, the sensitivity of detection can be enhanced evidently. Moreover, the simple biosensor can realize the detection of cancer cell-derived exosomes in one step without lengthy experimental operation and intricate design of nucleic acid sequences, holding great potential in the application for early clinical cancer diagnosis.

## Data Availability

All data presented in this study are available in the article and in Supplementary Materials.

## Supplementary Materials

The following supporting information can be downloaded at https://www.mdpi.com/article/10.3390/s23229139/s1: Table S1: DNA sequences used in this work; Figure S1: Optimization of the concentration of dual aptamers; Figure S2: Optimization of the volume ratio of Apt-CD63 to Apt-EpCAM; Figure S3: Optimization of the incubation time of exosomes.
