# Supplementary material for: Divalent Aptamer-Functionalized Nanochannels for Facile Detection of Cancer Cell-Derived Exosomes
Source: Sensors (Basel). 2023 Nov 13;23(22):9139. doi: 10.3390/s23229139 (PMC10674588; doi:10.3390/s23229139)
Supplement: Supplementary file 1 [file sensors-23-09139-s001.zip › sensors-2703775-supplementary.pdf]

Divalent Aptamer-Functionalized Nanochannels for Facile Detection of Cancer Cell-Derived Exosomes

Yue Huang [1], Fangfang Zhou [1], Fengjie Jia [2] and Nana Yang [3,*]

[1] Department of Food Science and Engineering, College of Light Industry and Food Engineering, Nanjing Forestry University, Nanjing 210037, China
[2] State Key Laboratory of Analytical Chemistry for Life Science, School of Life Sciences, Nanjing University, Nanjing 210023, China
[3] Department of Obstetrics and Gynecology, The First Affiliated Hospital of Nanjing Medical University, Nanjing 210029, China
* Correspondence: nanayang210@163.com

Table S1. DNA sequences used in this work.

Name	Sequence (5'-3')
Apt-CD63	CHO-$(CH_2)_6$-CACCCCACCTCGCTCCCGTGACACTAATGCTA
Apt-EpCAM	CHO-$(CH_2)_6$-CACTACAGAGGTTGCGTCTGTCCCACGTTGTCATGGGGGGTCCTG
Random DNA	CHO-$(CH_2)_6$-GGGGTGGGGTAGTGGTATGGAGCG

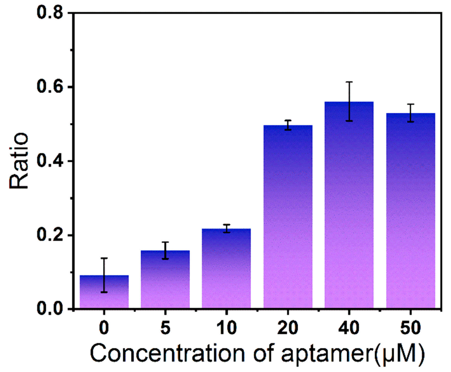

Figure S1. Change of current *vs* the concentration of dual aptamers. Ratio represents the value of $|(I - I_0)/I_0|$ (I_0 and I are the current values at −1.2 V in the absence and existence of HeLa cell-derived exosomes respectively). Error bars represent the standard deviations of three parallel measurements.

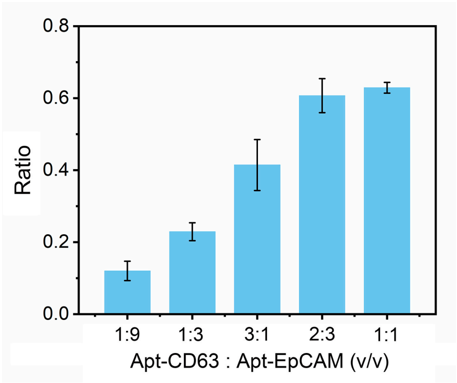

Figure S2. Change of current *vs* the volume ratio of Apt-CD63 to Apt-EpCAM. Ratio represents the value of $|(I - I_0)/I_0|$ (I_0 and I are the current values at −1.2 V in the absence and existence of HeLa cell-derived exosomes respectively). Error bars represent the standard deviations of three parallel measurements.

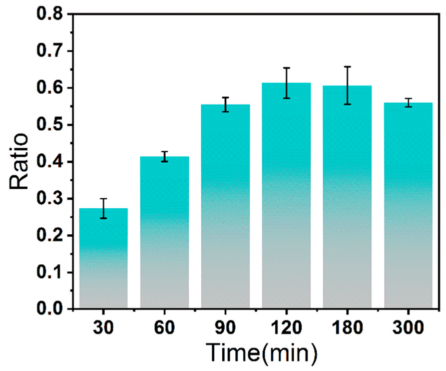

Figure S3. Change of current *vs* the incubation time of exosomes. Ratio represents the value of $|(I - I_0)/I_0|$ (I_0 and I are the current values at −1.2 V in the absence and existence of HeLa cell-derived exosomes respectively). Error bars represent the standard deviations of three parallel measurements.
